# Analysis of Physical Activity Using Wearable Health Technology in US Adults Enrolled in the All of Us Research Program: Multiyear Observational Study

**DOI:** 10.2196/65095

**Published:** 2024-12-10

**Authors:** Rujul Singh, Macy K Tetrick, James L Fisher, Peter Washington, Jane Yu, Electra D Paskett, Frank J Penedo, Steven K Clinton, Roberto M Benzo

**Affiliations:** 1 Division of Cancer Prevention and Control Department of Internal Medicine College of Medicine, The Ohio State University Columbus, OH United States; 2 Arthur G. James Cancer Hospital and Richard J. Solove Research Institute Columbus, OH United States; 3 Department of Information and Computer Sciences University of Hawaii at Manoa Honolulu, HI United States; 4 College of Medicine The Ohio State University Columbus, OH United States; 5 James Comprehensive Cancer Center The Ohio State University Columbus, OH United States; 6 Departments of Psychology and Medicine University of Miami Miami, FL United States; 7 Sylvester Comprehensive Cancer Center Miller School of Medicine University of Miami Miami United States; 8 Division of Medical Oncology Department of Internal Medicine The Ohio State University Columbus, OH United States

**Keywords:** Physical Activity Guidelines for Americans, accelerometry, All of Us Research Program, wearable activity monitors, health equity, multiyear activity tracking, activity intensity estimation, US adult population, sociodemographic determinants of physical activity, physical activity, wearables, United States, older adults, observational studies, longitudinal setting, sociodemographic determinants, physical activity data, Fitbit data, step-based method, adherence

## Abstract

**Background:**

To date, no studies have examined adherence to the 2018 Physical Activity Guidelines for Americans (PAGA) in real-world longitudinal settings using objectively measured activity monitoring data. This study addresses this gap by using commercial activity monitoring (Fitbit) data from the All of Us dataset.

**Objective:**

The primary objectives were to describe the prevalence of adherence to the 2018 PAGA and identify associated sociodemographic determinants. Additionally, we compared 3 distinct methods of processing physical activity (PA) data to estimate adherence to the 2008 PAGA.

**Methods:**

We used the National Institutes of Health’s All of Us dataset, which contains minute-level Fitbit data for 13,947 US adults over a 7-year time span (2015-2022), to estimate adherence to PAGA. A published step-based method was used to estimate metabolic equivalents and assess adherence to the 2018 PAGA (ie, ≥150 minutes of moderate- to vigorous-intensity PA per week). We compared the step-based method, the heart rate–based method, and the proprietary Fitbit-developed algorithm to estimate adherence to the 2008 PAGA.

**Results:**

The average overall adherence to the 2018 PAGA was 21.6% (3006/13,947; SE 0.4%). Factors associated with lower adherence in multivariate logistic regression analysis included female sex (relative to male sex; adjusted odds ratio [AOR] 0.66, 95% CI 0.60-0.72; *P*<.001); BMI of 25.0-29.9 kg/m^2^ (AOR 0.53, 95% CI 0.46-0.60; *P*<.001), 30-34.9 kg/m^2^ (AOR 0.30, 95% CI 0.25-0.36; *P*<.001), or ≥35 kg/m^2^ (AOR 0.13, 95% CI 0.10-0.16; *P*<.001; relative to a BMI of 18.5-24.9 kg/m^2^); being aged 30-39 years (AOR 0.66, 95% CI 0.56-0.77; *P*<.001), 40-49 years (AOR 0.79, 95% CI 0.68-0.93; *P*=.005), or ≥70 years (AOR 0.74, 95% CI 0.62-0.87; *P*<.001; relative to being 18-29 years); and non-Hispanic Black race or ethnicity (AOR 0.63, 95% CI 0.50-0.79; *P*<.001; relative to non-Hispanic White race or ethnicity). The Fitbit algorithm estimated that a larger percentage of the sample (10,307/13,947, 73.9%; 95% CI 71.2-76.6) adhered to the 2008 PAGA compared to the heart rate method estimate (4740/13,947, 34%; 95% CI 32.8-35.2) and the step-based method (1401/13,947, 10%; 95% CI 9.4-10.6).

**Conclusions:**

Our results show significant sociodemographic differences in PAGA adherence and notably different estimates of adherence depending on the algorithm used. These findings warrant the need to account for these disparities when implementing PA interventions and the need to establish an accurate and reliable method of using commercial accelerometers to examine PA, particularly as health care systems begin integrating wearable device data into patient health records.

## Introduction

The current Physical Activity Guidelines for Americans (PAGA) recommends 150 minutes of moderate-intensity aerobic physical activity (MPA), 75 minutes of vigorous-intensity aerobic physical activity (VPA), or a combination of moderate- and vigorous-intensity aerobic physical activity (MVPA) per week [1,2]. Adherence to these standards is linked to a 33% decline in all-cause mortality and provides health benefits including but not limited to decreased risk of hypertension, type 2 diabetes, cardiovascular disease, cancer, and dementia [2,3]. In addition, studies conducted on the All of Us (AoU) dataset have demonstrated additional associations between daily step count and decreased risk of obesity, gastroesophageal reflux disease, and sleep apnea [4]. The AoU program is a national initiative led by the National Institutes of Health (NIH) aimed at gathering health data (eg, survey, electronic medical record, and Fitbit wearable) from 1 million or more people in the United States to accelerate research and improve health outcomes through precision medicine.

Given the established health benefits of physical activity (PA), numerous studies have been conducted to evaluate PA patterns in US adults, including the frequency, intensity, and duration of PA [5-12]. However, these studies present several limitations. A significant number relied on self-report questionnaires [5,6,11,13,14]; evidence suggests that questionnaires are less robust in measuring PA and are susceptible to social desirability bias, recall bias, and seasonal response variation [15-21]. One approach to overcome this limitation is to use activity monitors, as activity monitors provide objective measures of movement and activity that are free from random and systemic errors that arise from self-report [22]. Most studies using such technology have collected less than 1 week’s worth of data [7,9,10,12]. Evidence indicates that 7-day monitoring periods typically result in intraclass correlations ≥80% while also capturing data from both weekdays and weekend days [22]. However, evidence also suggests that determining long-term habitual behavior patterns may require longer observation windows given significant seasonal and longitudinal variation in PA [19,22]. Compounding these limitations, activity-monitor estimates of PA intensity levels (ie, metabolic equivalents) have been determined in different ways (eg, using step count–, heart rate [HR]–, and proprietary device–generated estimates), which can lead to differing results [23,24]. As many health care systems are moving toward integrating these measures—many of which are “black box” outputs from commercial activity monitors like the Fitbit and Apple Watch—into patient health records [25,26], it is important to understand how these measures compare to one another in real-world observational settings.

Therefore, our observational study seeks to use the AoU dataset to address many of the aforementioned gaps and consists of 3 aims:

- Aim 1: Describe patterns of PA and adherence to the 2018 PAGA by sociodemographic variables (ie, sex, race or ethnicity, BMI, and age group)
- Aim 2: Evaluate the association of specific sociodemographic variables such as sex, race or ethnicity, BMI, and age group on the likelihood of meeting the 2018 PAGA
- Aim 3: Compare the proportion of adults meeting the 2008 PAGA as determined by 3 methods (step intensity, HR, and Fitbit proprietary algorithm)

## Methods

### Study Population

The AoU study is an NIH initiative to accelerate research and improve health outcomes through precision medicine by collecting a variety of data from various sources (eg, Fitbit, EHR, and survey) from adults in the United States. Under the AoU Fitbit Bring Your Own Device program, participants can link data (including data collected prior to the enrollment date) from their personal Fitbit device. A more detailed description of the AoU dataset can be found on the NIH AoU website [27]. The AoU program updates its dataset annually, and this study used the seventh version of the controlled tier dataset, released in April 2023. The study included 13,947 participants who consented to share their Fitbit data and possessed intraday HR and step count data for at least 1 valid week (requirements described below) of PA data from 2015 to 2022. All data periods prior to each participant’s 18th birthday were excluded from the sample.

### Ethical Considerations

Institutional review board approval was obtained from the AoU research program under protocol identification 2021-02-TN-001. Guidelines established by the NIH Office for Human Research Protections were followed, by which standardized protection of study participants’ rights and interests was ensured. A comprehensive informed consent procedure was implemented during study enrollment, whereby the right to discontinue involvement at any point was guaranteed to participants. Data security and participant confidentiality were safeguarded through multiple measures: secure data storage systems were implemented, access to identifying information was restricted, and confidentiality protocols were mandated through contractual agreements. Access to the Researcher Workbench was limited to authorized personnel by whom mandatory training had been completed and whose institutions were covered by active data use agreements. Compensation of US $25 was provided via a cash payment, gift card, or electronic voucher when biological specimens (blood, saliva, or urine) were collected at designated partner facilities. To protect participant privacy in accordance with AoU Research Program’s Data and Statistics Dissemination Policy, for groups with fewer than 20 participants, results were not presented to prevent potential identification.

### Measures

Minutes spent in sedentary, light-, moderate-, and vigorous-intensity activity were estimated using three distinct methods: (1) step intensity, (2) HR, and (3) a Fitbit proprietary algorithm. These outputs were then used to determine adherence to PAGA, where adherence was defined as meeting or exceeding a weekly average of 150 minutes of MVPA, where minutes of VPA count as 2 of MPA (ie, MPA+2×VPA≥150 minutes of MVPA). For aims 1 and 2, the step intensity–based method was used to estimate PA levels. The HR algorithm or equation (further described in the *PA Data Processing* section) was not used for various reasons that could confound our findings. First, the HR algorithm uses HR max as an input, and the formula used to calculate HR max has been shown to underestimate HR max in older ages [28]. Second, the accuracy of photoplethysmography sensors to measure HR is diminished in individuals with higher levels of melanin in the skin, which could confound the findings [29]. Third, equations that use HR max–based calculations (which is what was used in this analysis) to determine PA are poorly validated [30]. The Fitbit algorithm was not chosen as the primary method to estimate PA for 2 reasons. First, the algorithm only counts activity bouts of 10 minutes or more, which is aligned with the 2008 PAGA and not the current 2018 PAGA [31]. Second, the algorithm is not open source, which, from a scientific standpoint, limits transparency, reproducibility, peer review, collaboration, and improvement. In aim 3, we used all 3 algorithms to compare adherence to the 2008 PAGA, which only counts MVPA occurring in bouts of at least 10 minutes, where interruptions of up to 2 minutes were allowed in each bout (aligned with previous research) [9].

### Sociodemographic Classification

Participants were classified by sex (ie, male, female, and other or not specified), race or ethnicity (ie, Hispanic, non-Hispanic Asian or Pacific Islander, non-Hispanic White, non-Hispanic Black, and other or not specified), age group (18-29, 30-39, 40-49, 50-59, 60-69, and ≥70 years), and BMI (ie, normal: 18.5-24.9 kg/m^2^, overweight: 25.0-29.9 kg/m^2^, obese: 30-34.9 kg/m^2^, severely obese: ≥35 kg/m^2^, and other or not specified). Given that a 7-year collection period of data was used (2015-2022), some participants aged into the subsequent age group. In these instances, only data from the age grouping that had the most valid weeks were used in the analysis. BMI was determined by averaging all measurements taken between the first and the last valid week of data collection for each participant, and BMIs over 150 kg/m^2^ and under 12 kg/m^2^ were considered outliers and removed from the dataset (n=101 participants had 1 or more invalid BMI measurements removed using this criterion) [32]. Individuals with no recorded BMI measurements were grouped with the “other” category. Participants with BMIs under 18.5 kg/m^2^ were also grouped with the “other” category since there were fewer than 20 individuals with such measurements, and AoU restricts displaying data in such cases.

### PA Data Processing

Given the lack of sleep logs and the absence of accelerometry wear-log data typically used for classifying sleep or nonwear, the approach recommended by Claudel et al [33] was used, in which data from 11 PM to 5 AM were classified as sleep or nonwear and removed from the dataset. After the removal of sleep periods, valid wear days were defined as any 24-hour period (starting at midnight) with 10 or more hours of HR recordings and 100 or more steps, following conventionally defined guidelines [4,9,34,35]. All participants with at least 1 week of valid wear were included, where a valid week was defined as 3 valid days of wear within a 7-day interval (starting on January 1st of each year) [35].

**Figure 1:**
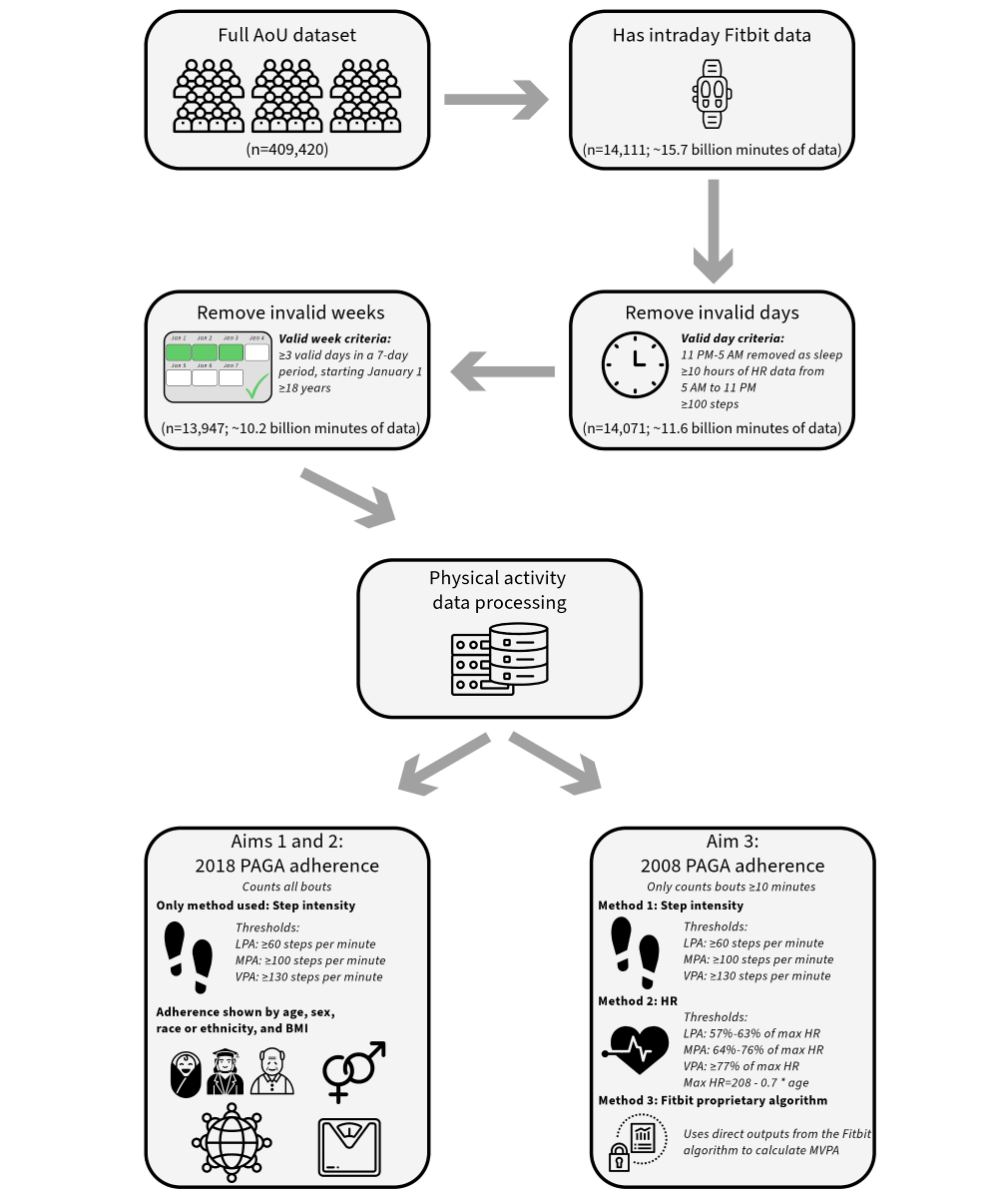
Data processing workflow. AoU: All of Us; HR: heart rate; LPA: light-intensity aerobic physical activity; MPA: moderate-intensity aerobic physical activity; MVPA: moderate- and vigorous-intensity aerobic physical activity; PAGA: Physical Activity Guidelines for Americans; VPA: vigorous-intensity aerobic physical activity.

Average weekly minutes spent sedentary in light-intensity aerobic physical activity (LPA), MPA, and VPA were calculated using three methods: (1) step intensity algorithm: a threshold was set at 60 steps per minute for LPA, at 100 steps per minute for MPA, and at 130 steps per minute for VPA [36]; (2) HR algorithm: a threshold was set at 57% of maximum HR for LPA, at 64% of maximum HR for MPA, and at 77% of maximum HR for VPA [37], and maximum HR was computed by the American College of Sports Medicine’s recommended formula of 208 – 0.7×age [38]; and (3) Fitbit algorithm: the device outputs of “sedentary,” “lightly active,” “fairly active,” and “very active” minutes were used as analogs for each of the 4 activity intensities defined in the 2018 PAGA [39]. Figure 1 shows the data processing workflow in detail.

The processing steps were used to determine valid weeks of data and to determine adherence to the 2008 and 2018 PAGA.

### Statistical Analysis

A CONSORT (Consolidated Standards of Reporting Trials) diagram was prepared to show the number of individuals excluded from the dataset and can be found in Multimedia Appendix 1. We present descriptive summary statistics for our study sample using mean and SDs for continuous characteristics as well as frequencies for categorical variables.

Our research aim 1 was to describe PA levels and adherence to the 2018 PAGA across sociodemographic groups. To describe PA levels in our sample, we illustrate daily step counts, average weekly MVPA minutes, and IQRs by sex and age as well as sex and BMI. To describe the adherence of our sample to the guidelines, we present the proportion of participants meeting the 2018 PAGA (and 95% Wald CIs) [40] by sex, race or ethnicity, BMI, and age group. Significant differences in proportions were assessed using pairwise chi-square tests with a Bonferroni-adjusted α level of 0.0012 (43 separate pairwise comparisons were considered) [41].

Our research aim 2 was to evaluate the association of specific sociodemographic variables with the likelihood of meeting the 2018 PAGA. Univariate and multivariate logistic regression were used to identify demographic factors associated with meeting the 2018 PAGA. Multivariate analysis controlled for age group, sex, BMI, and race or ethnicity, as these factors have been shown to have strong associations with PA (selected a priori) [42]. Linearity of logits was tested via examination of component residual plots, and multicollinearity was tested via the computation of variance inflation factors for each coefficient (with all variance inflation factor values confirmed to be under 5) [43]. Odds ratios (ORs) and 95% Wald CIs were reported for each coefficient. Significance was assessed using 2-sided Wald tests for significance on each coefficient, with an α level of 0.05 and 2 to 5 degrees of freedom for the univariate model (depending on the variable being tested) and 16 degrees of freedom for the multivariate model [40].

Our research aim 3 was to compare 3 different methods (step intensity, HR, and Fitbit proprietary) for estimating the proportion of individuals meeting the 2008 PAGA. To assess differences in proportions meeting guidelines as determined by each method, 2-sided pairwise McNemar tests were performed with a Bonferroni-adjusted α level of .0008, given the paired nature of the data (63 separate pairwise comparisons were considered) [44]. All analyses were performed using R (version 4.4.0; R Foundation for Statistical Computing) and RStudio (version 2024.4.0.735; Posit PBC) within the AoU Researcher Workbench.

## Results

### Participant Sociodemographic Characteristics

A total of 14,111 participants had intraday Fitbit HR, activity summary, and step count data available within the dataset (Table 1). After processing and removing for nonwear, the dataset included 13,947 individuals with over 1.6 million total weeks of data (averaging ~115 weeks of data collection for each individual participant). Our sample was predominantly female (9533/13,947, 68.5%) and identified predominantly as non-Hispanic White (11,109/13,947, 79.7%). The average age was 50.8 (SD 15.8) years, and the average BMI was 29.4 (SD 6.7) kg/m^2^; nearly half (6590/13,947, 47.3%) of participants did not have valid BMI measurements available.

**Table 1:** Descriptive summary of the study sample^a^.

Variables				Weeks of included data (n=1,607,789), n		Participants (n=13,947), n (%)
**Sex**						
		Male		487,069		4007 (28.7)
		Female		1,074,521		9553 (68.5)
		Other or not specified		46,199		387 (2.8)
**Race** **or** **ethnicity**						
		Hispanic		32,695		310 (2.2)
		Non-Hispanic Asian or Pacific Islander		47,683		430 (3.1)
	Non-Hispanic Black		71,482		684 (4.9)	
	Non-Hispanic White		1,311,659		11,109 (79.7)	
		Two or more races		31,603		293 (2.1)
		Other or not specified		112,667		1121 (8)
**BMI (kg/m^2^)^b,c^**						
		18.5-24.9		276,329		1994 (14.3)
		25-29.9		342,679		2487 (17.8)
		30-34.9		203,851		1501 (10.8)
		≥35		165,549		1375 (9.9)
		Other or not specified		619,381		6590 (47.3)
**Age group (years)^d^**						
		18-29		141,806		1511 (10.8)
		30-39		274,727		2528 (18.1)
		40-49		255,711		2259 (16.2)
		50-59		322,264		2760 (19.8)
		60-69		387,915		3102 (22.2)
		≥70		225,366		1787 (12.8)

^a^Data include all weeks of valid data from all participants, ranging from 2015 to 2022.

^b^BMI was calculated as the average of all measured values measured between the first and last valid weeks for each participant. If no measured values were available, BMI was categorized as “other or not specified.” Individuals with a BMI under 18.5 kg/m^2^ are categorized as “other or not specified” due to the small sample size.

^c^Mean BMI 50.8, SD 15.8 kg/m^2^.

^d^Mean age 29.4, SD 6.7 years.

### Aim 1: Descriptive Analysis of PA

In total, 21.6% (3006/13,947) of individuals in the study sample met the 2018 PAGA, as determined by the step-intensity algorithm (Table 2). The average weekly minutes spent in MPA was 80.8 (SE 0.7) minutes, in VPA was 10.3 (SE 0.3) minutes, in MVPA (MPA+2×VPA) was 101.4 (SE 1.0) minutes, and the average daily step count was 7758 (SE 28) steps.

**Table 2:** Analysis of sociodemographic factors associated with meeting 2018 Physical Activity Guidelines for Americans (PAGA)^a^.

		Total participants, n	Participants with MVPA^b^ from step counts			Univariate regression			Multivariate regression^c^	
			n (%)	95% CI (%)	SE (%)	Odds ratio (95% CI)	*P* value	Multivariate odds ratio (95% CI)		*P* value
**Total**		13,947	3006 (21.6)	20.8-22.4	0.4	—^d^	—	—		—
**Sex**										
	Male	4007	1069 (26.7)	24.7-28.7	1.0	—	—	—		—
	Female	9553	1864 (19.5)	18.5-20.5	0.5	*0.67 (0.61-0.73)* ^e^	<.001	*0.66 (0.60-0.72)*		<.001
	Other or not specified	387	73 (18.9)	14.0-23.8	2.5	*0.64 (0.49-0.83)*	<.001	0.75 (0.55-1.01)		.07
**Race or ethnicity**										
	Hispanic	310	60 (19.4)	13.9-24.9	2.8	0.85 (0.63-1.12)	.25	0.88 (0.65-1.17)		.39
	Non-Hispanic Asian or Pacific Islander	430	135 (31.4)	24.9-37.9	3.3	*1.61 (1.31-1.98)*	<.001	*1.35 (1.08-1.67)*		.007
	Non-Hispanic Black	684	87 (12.7)	9.8-15.6	1.5	*0.51 (0.41-0.64)*	<.001	*0.63 (0.50-0.79)*		<.001
	Non-Hispanic White	11,109	2454 (22.1)	21.1-23.1	0.5	—	—	—		—
	Two or more races	293	61 (20.8)	14.9-26.7	3.0	0.93 (0.69-1.23)	.60	0.96 (0.71-1.28)		.79
	Other or not specified	1121	209 (18.6)	15.9-21.3	1.4	*0.81 (0.69-0.94)*	.008	0.84 (0.70-1.01)		.07
**BMI (kg/m^2^)^f^**										
	18.5-24.9	1994	727 (36.5)	33.2-39.8	1.7	—	—	—		—
	25-29.9	2487	598 (24.0)	21.8-26.2	1.1	*0.55 (0.48-0.63)*	<.001	*0.53 (0.46-0.60)*		<.001
	30-34.9	1501	224 (14.9)	12.7-17.1	1.1	*0.31 (0.26-0.36)*	<.001	*0.30 (0.25-0.36)*		<.001
	≥35	1375	88 (6.4)	5.0-7.8	0.7	*0.12 (0.09-0.15)*	<.001	*0.13 (0.10-0.16)*		<.001
	Other or not specified	6590	1369 (20.8)	19.6-22.0	0.6	*0.46 (0.41-0.51)*	<.001	*0.45 (0.41-0.51)*		<.001
**Age group (years)**										
	18-29	1511	389 (25.7)	22.8-28.6	1.5	—	—	—		—
	30-39	2528	446 (17.6)	15.8-19.4	0.9	*0.62 (0.53-0.72)*	<.001	*0.66 (0.56-0.77)*		<.001
	40-49	2259	426 (18.9)	16.9-20.9	1.0	*0.67 (0.57-0.78)*	<.001	*0.79 (0.68-0.93)*		.005
	50-59	2760	583 (21.1)	19.1-23.1	1.0	*0.77 (0.67-0.90)*	.001	0.88 (0.76-1.03)		.10
	60-69	3102	773 (24.9)	22.9-26.9	1.0	0.96 (0.83-1.10)	.54	1.00 (0.86-1.16)		.97
	≥70	1787	389 (21.8)	19.4-24.2	1.2	*0.80 (0.68-0.94)*	.007	*0.74 (0.62-0.87)*		<.001

^a^The table shows the proportion of individuals meeting the 2018 Physical Activity Guidelines for Americans (PAGA) aerobic activity guidelines and results from logistic univariate and multivariate regression analyses used to show the impact of various demographic factors on the probability of meeting the 2018 PAGA.

^b^MVPA: moderate- and vigorous-intensity aerobic physical activity.

^c^Multivariate regression adjusts for sex, race or ethnicity, BMI, and age.

^d^Not applicable (reference groups).

^e^Italicized odds ratios (95% CI) reflect *P* values less than .05.

^f^BMI was calculated as the average of all measured values measured between the first and last valid weeks for each participant. If no measured values were available, BMI was categorized as “other or not specified.” Individuals with a BMI under 18.5 kg/m^2^ are categorized as “other or not specified” due to the small sample size.

**Figure 2:**
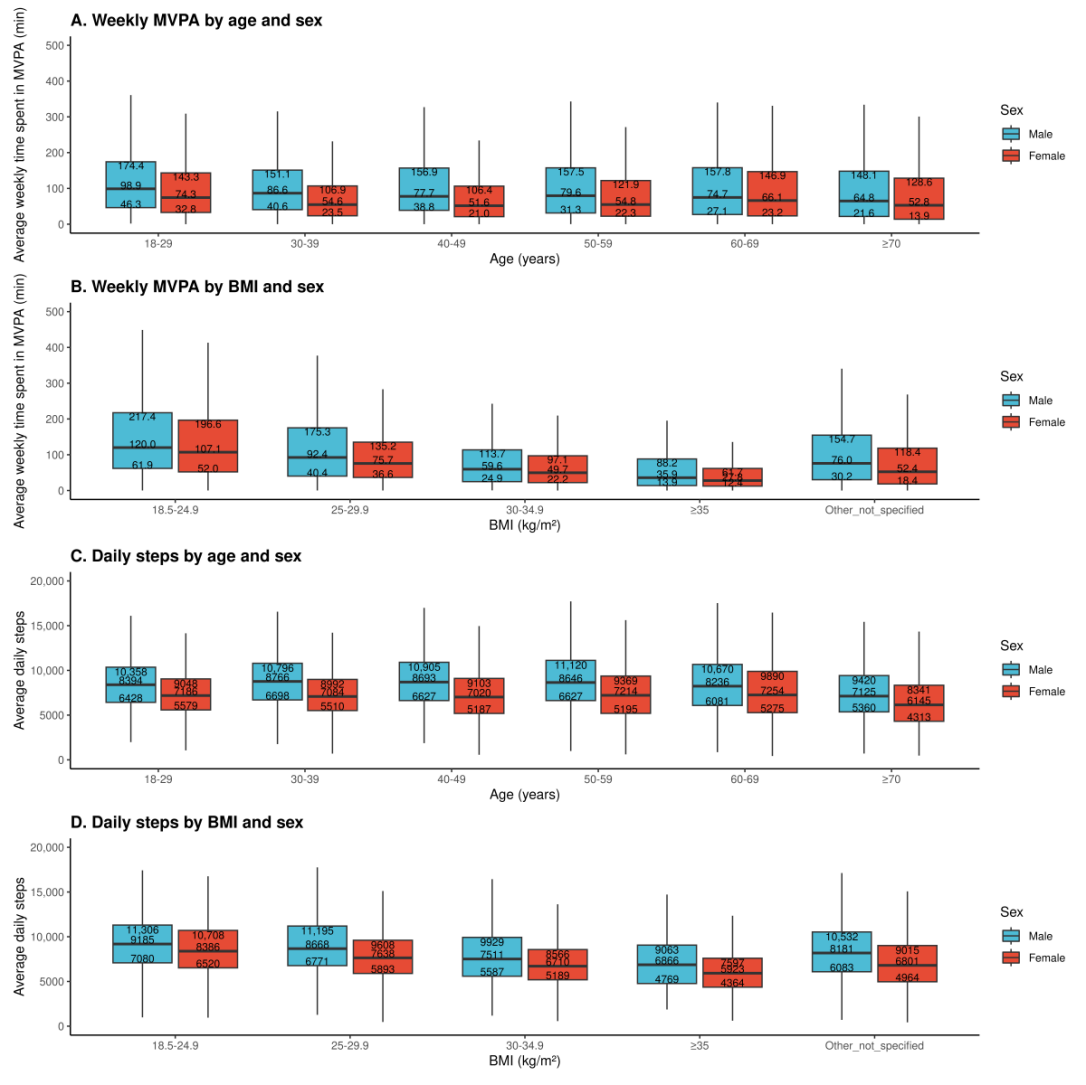
Weekly minutes of MVPA (computed via step count algorithm) and daily step counts across sex, age, and BMI. Total weekly minutes of MVPA are defined as minutes of moderate activity + 2×minutes of vigorous activity, shown segmented by (A) age and sex and (B) BMI and sex. Daily step counts are shown segmented by (C) age and sex and (D) BMI and sex. All 4 panels show 25th percentile, median, and 75th percentile values in box plot format. The lower whisker extends to the smallest value, which is greater than or equal to the 25th percentile value – 1.5×IQR. The upper whisker extends to the largest value, which is less than or equal to the 25th percentile value + 1.5×IQR. BMI was calculated as the average of all measured values between the first and last valid weeks for each participant. If no measured values were available, BMI was categorized as “other or not specified.” Individuals with a BMI under 18.5 kg/m2 are categorized as “other or not specified” due to the small sample size. MVPA: moderate- and vigorous-intensity aerobic physical activity.

We found that male individuals were more likely than female individuals to meet PAGA (1069/4007, 26.7% vs 1864/9553, 19.5%, respectively; *P*<.0001). Among racial or ethnic groups, non-Hispanic Asian or Pacific Islander individuals had higher adherence compared to Hispanic (135/430, 31.4% vs 60/310, 19%, respectively; *P*=.0003), non-Hispanic White (2454/11,109, 22.1%; *P*<.0001), and non-Hispanic Black (87/684, 13%; *P*<.0001) individuals. Non-Hispanic White individuals also had higher adherence than non-Hispanic Black (*P*<.0001) individuals. Regarding BMI, there were significant differences in adherence across normal weight (727/1994, 36.5%), overweight (598/2487, 24%), obese (224/1501, 14.9%), and severely obese groups (88/1375, 6.4%; *P*<.0001 for each pairwise comparison), with adherence decreasing as BMI increased. In terms of age, the 18-29 years age group showed the highest adherence (389/1511, 25.7%), significantly higher than the 30-39 years age group (446/2528, 17.6%; *P*<.0001), 40-49 years age group (426/2259, 18.9%; *P*<.0001), and 50-59 years age group (583/2760, 21.1%; *P*=.0007). The 30-39 years age group adhered less than both the 60-69 years age group (773/3102, 24.9%; *P*<.0001) and ≥70 years age group (389/1787, 21.8%; *P*=.0008). The 40-49 years age group and 50-59 years age group both had lower adherence in comparison to the 60-69 years age group (*P*<.0001 and *P*=.0007, respectively). A more detailed breakdown of the average time spent in MVPA by sex and age group and by sex and BMI is presented in Figure 2. Similarly, the average daily step count by age group and sex and by sex and BMI is also presented in Figure 2. Multimedia Appendix 2 presents minutes per week (means and SEs) of LPA, MPA, VPA, and MVPA among US adults by sex, age group, ethnicity, and BMI.

### Aim 2: Sociodemographic Influences on Adherence to the 2018 PAGA

Univariate and multivariate logistic regression analyses identified sex, race or ethnicity, BMI, and age as significantly associated with meeting the 2018 PAGA at the 0.05 α level, as indicated by 2-sided tests (Table 2). Univariate analysis indicated that female individuals were significantly less likely than male individuals to meet the 2018 PAGA, with a univariate OR of 0.67 (95% CI 0.61-0.73). Non-Hispanic Black individuals also had a lower likelihood of adherence when compared to non-Hispanic White individuals, with a univariate OR of 0.51 (95% CI 0.41-0.64). Conversely, non-Hispanic Asian individuals were more likely to meet the guidelines than non-Hispanic White individuals in univariate analysis (OR 1.61, 95% CI 1.31-1.98). Higher BMI was inversely associated with decreased PA, and the univariate analysis found significant differences in adherence across all age groups except the 60-69 years age group compared to the 18-29 years reference group.

The multivariate analysis largely agreed with the univariate analysis. Both female and non-Hispanic Black individuals were less likely to meet the 2018 PAGA guidelines than their reference groups, with multivariate ORs of 0.66 (95% CI 0.60-0.72) and 0.63 (95% CI 0.50-0.79), respectively. Multivariate analysis also indicated that non-Hispanic Asian individuals were more likely to meet the guidelines than non-Hispanic White individuals, with an OR of 1.35 (95% CI 1.08-1.67). As with the univariate analysis, higher BMI was inversely associated with decreased PA. However, in multivariate analysis, only the 30-39, 40-49, and ≥70 years age groups showed significant differences compared to the reference group (18-29 years age group).

### Aim 3: Evaluating 3 Methods for Assessing Compliance With the 2008 PAGA

All 3 methods (step intensity, HR, and Fitbit proprietary) showed significant differences in the total percentage of individuals meeting the 2008 PAGA, with *P* values less than .0001 for each comparison. The Fitbit algorithm indicated the highest adherence in our sample (10,307/13,947, 73.9%; 95% CI 71.2-76.6), followed by the HR-based algorithm (4740/13,947, 34%; 95% CI 32.8-35.2) and then step intensity–based algorithm (1401/13,947, 10%; 95% CI 9.4-10.6; Table 3). All pairwise comparisons across the 3 algorithms by each sociodemographic variable also showed significant differences (*P*<.0001 for each comparison), with 1 notable exception: for the 18-29 years age group, the HR method and the step-intensity algorithm were not statistically significantly different (*P*=.39).

**Table 3:** Percentage of US adults who meet 2008 physical activity guidelines based on Fitbit proprietary algorithm, heart rate (HR), and step counts from accelerometer data^a^.

		Total participants, n	Participants with MVPA^b^ from algorithm				Participants with MVPA from HR values				Participants with MVPA from step counts		
			n (%)	95% CI^c^ (%)	SE (%)	n (%)		95% CI^c^ (%)	SE (%)	n (%)		95% CI^c^ (%)	SE (%)
**Total**		13,947	10,307 (73.9)	71.2-76.6	1.4	4740 (34.0)		32.8-35.2	0.6	1401 (10.0)		9.4-10.6	0.3
**Sex**													
	Male	4007	3600 (89.8)	80.6-99.0	4.7	1476 (36.8)		24.4-39.2	1.2	544 (13.6)		12.4-14.8	0.6
	Female	9553	6425 (67.3)	64.4-70.2	1.5	3115 (32.6)		31.2-34.0	0.7	822 (8.6)		8.0-9.2	0.3
	Other or not specified	387	282 (72.9)	56.6-89.2	8.3	149 (38.5)		30.7-46.3	4.0	35 (9.0)		5.9-12.1	1.6
**Race** **or** **ethnicity**													
	Hispanic	310	223 (71.9)	54.1-89.7	9.1	66 (21.3)		15.4-27.2	3.0	23 (7.4)		4.3-10.5	1.6
	Non-Hispanic Asian or Pacific Islander	430	360 (83.7)	62.3-100.0	10.9	124 (28.8)		22.7-34.9	3.1	56 (13.0)		9.3-16.7	1.9
	Non-Hispanic Black	684	428 (62.6)	53.0-77.2	4.9	232 (33.9)		28.6-39.2	2.7	41 (6.0)		4.0-8.0	1.0
	Non-Hispanic White	11,109	8302 (74.7)	71.6-77.8	1.6	3913 (35.2)		33.8-36.6	0.7	1185 (10.7)		10.1-11.3	0.3
	Two or more races	293	200 (68.3)	51.4-85.2	8.6	61 (20.8)		14.9-26.7	3.0	<20 (6.8)		N/A^d^	1.3
	Other or not specified	1121	794 (70.8)	61.6-80.0	4.7	344 (30.7)		26.8-34.6	2.0	83 (7.4)		5.8-9.0	0.8
**BMI (kg/m** ^**2**^ **)** ^e^													
	18.5-24.9	1994	1647 (82.6)	73.0-92.2	4.9	879 (44.1)		40.2-48.0	2.0	364 (18.3)		16.1-20.5	1.1
	25-29.9	2487	2034 (81.8)	73.6-90.0	4.2	986 (39.6)		36.5-42.7	1.6	275 (11.1)		9.7-12.5	0.7
	30-34.9	1501	1109 (73.9)	65.5-82.3	4.3	463 (30.8)		27.5-34.1	1.7	96 (6.4)		5.0-7.8	0.7
	≥35	1375	856 (62.3)	55.4-69.2	3.5	324 (23.6)		20.7-26.5	1.5	25 (1.8)		1.0-2.6	0.4
	Other or not specified	6590	4661 (70.7)	67.0-74.4	1.9	2088 (31.7)		30.1-33.3	0.8	641 (9.7)		8.9-10.5	0.4
**Age group (years)**													
	18-29	1511	1106 (73.2)	64.8-81.6	4.3	119 (7.9)		6.3-9.5	0.8	108 (7.1)		5.7-8.5	0.7
	30-39	2528	1798 (71.1)	65.0-77.2	3.1	323 (12.8)		11.2-14.4	0.8	161 (6.4)		5.4-7.4	0.5
	40-49	2259	1570 (69.5)	63.2-75.8	3.2	547 (24.2)		21.8-26.6	1.2	188 (8.3)		7.1-9.5	0.6
	50-59	2760	2022 (73.3)	67.0-79.6	3.2	1068 (38.7)		35.8-41.6	1.5	285 (10.3)		9.1-11.5	0.6
	60-69	3102	2431 (78.4)	71.7-85.1	3.4	1639 (52.8)		49.1-56.5	1.9	428 (13.8)		12.4-15.2	0.7
	≥70	1787	1380 (77.2)	68.6-85.8	4.4	1044 (58.4)		52.9-63.9	2.8	231 (12.9)		11.1-14.7	0.9

^a^This table shows the proportion of individuals meeting the 2008 Physical Activity Guidelines for Americans (PAGA) aerobic activity guidelines of 150 minutes a week of moderate to vigorous physical activity. Only activity bouts ≥10 minutes in duration included in determining physical activity.

^b^MVPA: moderate- and vigorous-intensity aerobic physical activity.

^c^95% Wald CIs shown for the proportion of each demographic group meeting the 2008 PAGA aerobic activity guidelines. CIs capped at a maximum value of 100%.

^d^All of Us policy prevents the display of any participant count less than 20.

^e^BMI was calculated as the average of all measured values measured between the first and last valid weeks for each participant. If no measured values were available, BMI was categorized as “other or not specified.” Individuals with a BMI under 18.5 kg/m^2^ are categorized as “other or not specified” due to the small sample size.

## Discussion

### Principal Findings

We found that approximately one-fifth (3006/13,947, 21.6%) of our sample met the 2018 PAGA using the step count algorithm, and the average minutes of MVPA per week (101.4 minutes) was nearly 50 minutes lower than recommended. After controlling for confounders, we found that several demographic factors were independently associated with meeting PA guidelines. Women were less likely to meet PA guidelines than men, and higher BMI was shown to be inversely associated with adhering to the 2018 PAGA. Race or ethnicity and age also showed strong associations, as non-Hispanic Black individuals were less likely than non-Hispanic White individuals to meet guidelines, whereas non-Hispanic Asian individuals were more likely to meet guidelines. Interestingly, age showed a more nuanced relationship with meeting guidelines, with PA declining until middle age, then increasing briefly, before declining again in old age (≥70 years) when compared to the 18-29 years reference group.

### Comparison With Prior Work

Our report showed substantially lower proportions of participants meeting the 2018 PAGA compared to a report completed by the National Center for Health Statistics (NCHS; which showed that ~47% of US adults met the latest aerobic PAGA in 2020; N=31,568) [45] but higher proportions compared to previous studies completed by Tucker et al [10] (N=3082) as well as Troiano et al [9] (N=6329), which evaluated adherence to the 2008 PAGA and averaged around 5%. The lower proportion in comparison to NCHS reports was likely explained by response bias in surveys, as surveys have been shown to result in higher proportions of reported PA compared to objective measures [15,16]. In addition, studies have also shown that individuals are more likely to overreport PA for social acceptance reasons [46]. Finally, pedometer-measured PA may not capture activities that exclude or limit stepping (eg, yoga, tai chi, bicycling, and swimming), thus limiting the ability to accurately estimate PA levels [46]. Conversely, we observed higher proportions of participants meeting the PAGA in comparison to earlier studies using activity monitor data. We hypothesize that this may be caused by several factors. First, many previous studies measured adherence to a previous iteration of the 2008 PAGA, where only bouts of 10-minute MVPA are included [9,10]. Aligned with this observation, our estimates of adherence fall to 10% (95% CI 9.4-10.6) when following the 2008 PAGA 10-minute bout requirement. Second, selection bias is also likely to have occurred in this study; since individuals were self-selecting to participate in the AoU study and had chosen to purchase activity monitors of their own volition, they may have been more likely to be physically active compared to randomly sampled US adults (eg, National Health and Nutrition Examination Survey). Third, the convenience sampling strategy of our study likely resulted in a sample distribution that was not demographically reflective of the general US population. Finally, while prevalence estimates remain low, longitudinal studies have shown an increase in aerobic PA over time, as general PA levels have increased over the past 30 years [5]. Given that it has been over a decade since accelerometers have been used in the National Health and Nutrition Examination Survey [47], there is a unique opportunity to recreate this approach in a nationally representative sample, which could allow us to gain further insights into this large Fitbit database.

When evaluating sociodemographic associations, our findings align with previous studies regarding the relationship between sex, BMI, and PA. This alignment was observed both in self-reported data and data derived from accelerometry [9,10,12,45,48]. We found that women were significantly less physically active than men. Although further research is needed to fully understand this phenomenon, existing studies suggest that various factors contribute to lower PA participation among women. These factors include lower levels of motivation to participate in PA, diminished levels of peer and early parental social support for PA, and societal expectations that encourage women to prioritize other responsibilities, such as domestic duties and childcare [49]. We also found that higher BMI was inversely associated with PA. This finding also aligns with both self-reported data and data derived from accelerometry [5,7,12,48]. This phenomenon has been well studied; prior studies have established that higher levels of PA cause a reduction in BMI and that higher BMI also serves as a barrier to PA participation [50,51].

For age, our study showed slightly different associations compared to prior studies. Prior cross-sectional studies have shown a negative correlation between increased age and PA at all age ranges [8-12,48]. However, our study showed a decline in PA when compared to a reference group aged 18-29 years until age 50 years, at which point, PA increased back to proportions seen in young adulthood, then declined again after age 70 years. We hypothesize several possible mechanisms of action for this phenomenon. Some targeted studies focusing on older adults have shown an increase in leisure time PA following retirement. Given the 60-69 years age group falls into retirement age, this may play a factor in a temporary resurgence of PA [52]. Studies have also shown a lower proportion of PA among adults with children versus child-free adults. As children leave their households, individuals may have more time and energy to exercise [53].

Regarding race, we found that non-Hispanic Black individuals were less likely to meet the 2018 PAGA guidelines compared to non-Hispanic White individuals. This finding is consistent with most prior studies [10,54,55], which demonstrated similar racial disparities in PA levels using both objective measures (eg, accelerometry) [10] and subjective self-report methods [54,55]. Tucker et al [10] evaluated adherence to the 2008 PAGA using accelerometry data (N=4773) and found that 8% (1.40% SE) of non-Hispanic Black individuals adhered to the 2008 PAGA while 10.1% (1.16% SE) of non-Hispanic White individuals adhered to the 2008 PAGA (statistical tests for significance were not conducted). Wilson-Frederick et al [54] found in a national survey–based study (N=67,790) that Black individuals had higher adjusted odds of physical inactivity (with physical inactivity defined as 0 minutes of weekly MVPA) compared to White individuals after controlling for confounders (adjusted odds ratio [AOR] 1.40, 95% CI 1.30-1.51). Similarly, a Centers for Disease Control and Prevention report using self-report data from the Behavioral Risk Factor Survey System administered from 2017 through 2020 (N=~1.6 million, exact sample size not disclosed) also showed that non-Hispanic Black individuals had a 30% prevalence of physical inactivity outside of work compared to 23% for non-Hispanic White individuals (statistical tests for significance were not conducted) [55]. In contrast, Matthews et al [11], in a 2019 study (N=2640), found no significant differences in daily PA levels between non-Hispanic Black and non-Hispanic White individuals in a study where PA was self-reported. The divergence between our findings and those of Matthews et al [11] may be attributed to methodological differences, including their smaller sample size, their use of 24-hour survey recalls administered on a single day (which have been shown to result in biased estimates of MVPA at both group and individual level in adults) [19,56,57], and the Activities Collected Over Time Over 24-hours survey instrument used in their study, which has not been validated across all race and ethnic groups investigated.

Our findings showed that non-Hispanic Asian individuals were more likely to adhere to the 2018 PAGA when compared to non-Hispanic White individuals. Although few objective accelerometry-based studies evaluating PA prevalence in Asian American individuals have been conducted, our findings align with a Centers for Disease Control and Prevention report using self-report data from the Behavioral Risk Factor Survey System administered from 2017 through 2020 (N=~1.6 million, exact sample size not disclosed). This report found that non-Hispanic Asian individuals had a 20.1% prevalence of physical inactivity outside of work compared to 23% for non-Hispanic White individuals when measured using surveys (statistical tests for significance were not conducted). In contrast to our findings, Stella et al [58] conducted a survey study in 2 major US cities (Los Angeles: N=17,462 and New York City: N=8036) and found that non-Hispanic White individuals were more likely than non-Hispanic Asian individuals to adhere to the 2018 PAGA guidelines after adjusting for confounders (AOR 1.35, 95% CI 1.09-1.68 and AOR 1.45, 95% CI 1.13-1.86, respectively). The divergence between our findings and those of Stella et al [58] may be attributed to methodological differences, including their use of subjective PA measures, which are generally less accurate than objective methods [15-18,20,21], and their study’s exclusive focus on large metropolitan areas. We theorize that family structures and dynamics in Asian households may contribute to the higher PA rates observed among non-Hispanic Asian individuals in our study, although additional research is needed to confirm this relationship. Prior studies have suggested that single-parent households lead to reductions in PA participation for children [59], that childhood PA behavior patterns are strongly predictive of PA patterns in adulthood [60], and that middle-aged adults with strong family support networks are more likely to engage in PA [61]. Asian American individuals have among the lowest single-parentage rates and the highest proportion of individuals living in intergenerational family households, which may play a role in influencing lifelong PA behavior patterns [62].

When comparing the Fitbit proprietary algorithm, the HR-based algorithm, and the step intensity–based algorithm, we found significant discrepancies in measuring adherence to the 2008 PAGA. The Fitbit proprietary algorithm indicated the highest adherence proportion at 73.9% (10,307/13,947), followed by the HR algorithm at 34% (4740/13,947) and the step intensity algorithm at 10% (1401/13,947). Notably, the Fitbit proprietary algorithm showed a nearly 7-fold higher proportion of individuals meeting the 2008 PAGA compared to the step-intensity algorithm.

Our findings regarding the Fitbit algorithm align with prior validation studies, which have demonstrated consistent overestimation of MVPA compared to validated accelerometry-based methods in free-living environments [39,63-65]. The degree of overestimation has been estimated to be as much as an additional hour of MVPA per day, which could lead to notable changes in the estimated prevalence of adherence to PAGA [39]. This overestimation is concerning, as it could also lead to a significant portion of the US population being incorrectly classified as meeting PA guidelines. Consequently, these individuals might not be targeted for necessary PA interventions, potentially increasing their risk for adverse health outcomes. Furthermore, evidence suggests that the degree of overestimation increases as the volume of MVPA increases, suggesting that the accuracy of MVPA measurement is further reduced in active individuals [39]. Rosenberger et al [63] conducted a study to compare Fitbit against ActiGraph measures in a 24-hour wear protocol and found that Fitbit’s estimates of daily MVPA had a mean absolute percentage error greater than 60% compared to the Actigraph [63]. Similarly, Feehan et al [65] found in a systematic review comparing Fitbit devices against validated Actical and ActiGraph activity monitors that the median difference in time spent in light to vigorous PA ranged from 52% to 390% across 8 separate studies. This overestimation has been found to occur in both wrist-worn and hip-worn Fitbit devices [64]. Given the increasing adoption of Fitbit devices in both the general population and clinical settings, it is crucial to accurately assess their measurement precision and identify the factors contributing to any inaccuracies.

Similarly, the HR-based algorithm also showed meaningfully higher estimates of PA than the step intensity–based algorithm. We hypothesize that this finding may primarily be due to two reasons. First, the commonly used formula for calculating maximum HR tends to systematically underestimate the maximum HR in older individuals, leading to a systemic upward bias in PA measurements for this demographic [28]. Second, the HR algorithm ignores the impact of sex on determining maximum HR, which may result in systemic overestimation of PA in women since women have higher HRs than men [66]. Overestimation of PA levels is a problem because it can lead to the underidentification of individuals who may benefit from increased activity, thus increasing their risk of a plethora of adverse health events. This is particularly important, as Fitbit and other commercial activity monitors are being integrated into the electronic medical record to support physician monitoring of patient health and inform medical decisions [25,67].

### Strengths and Limitations

The principal strength of this study is the extensive size of the sample. We analyzed an unprecedented 1.6 million weeks of data collected over 7 years, involving over 13,000 participants. This extensive dataset enabled us to examine sociodemographic associations with adherence to PA guidelines and to assess differences in algorithms for measuring PA in an ecologically valid manner. This ecological validity was further strengthened by our ability to use objective, accelerometry-derived measures rather than self-reported data to analyze PA. Finally, the paired nature of the data—comprising MVPA as measured by Fitbit, step count, and HR—enabled a statistically robust analysis across a very large dataset to compare the outcomes from each algorithm. However, this study also presents several limitations. First, the “Bring Your Own Device” approach of the AoU program likely resulted in a sample that is not nationally representative of US adults. While our analysis controlled for age, race, BMI, and sex, we did not address potential bias arising from participants possibly being more active due to owning activity monitors. Although the AoU program is piloting initiatives to distribute Fitbit devices to increase sample diversity, this information is not yet publicly available. Second, the sample was not reweighted to reflect demographic characteristics for the general American population. It is important to note that although our multivariate analysis controlled for sex, race or ethnicity, BMI, and age, the raw adherence proportions shown do not represent the target proportions, as our population was older, White, and more female compared to the general US population. Third, participants used various Fitbit models with differing firmware, which may have introduced measurement discrepancies due to differences in device and proprietary analyses. Fourth, the analysis did not control for confounding by income or educational status, which have been shown to be associated with PA levels [42]. Fifth, although the step-intensity method was chosen as the most accurate method for estimating PA and determining the 2018 PAGA adherence when using Fitbit devices, the accuracy of the device at measuring step counts degrades during activities involving slow ambulation and in individuals with mobility limitations [65]. Finally, we could not accurately remove sleep time from the wear log since we did not have sleep diaries, minute-level accelerometry count data, or validated algorithms that could be used to determine sleep times using Fitbit data. Future research should consider using sleep logs or raw accelerometry data to assess PA levels more accurately.

### Conclusions

Overall, our study resulted in three primary conclusions: (1) adherence proportions to the 2018 PAGA are lower than indicated by NCHS reports and differ by sociodemographic classification; (2) key sociodemographic characteristics, including age, BMI, race, and sex, are strongly correlated with adherence proportions to PA guidelines as outlined by PAGA; and (3) various algorithms based on proprietary Fitbit data, HR, and step count produce significantly different outcomes in measuring PA. These findings have important implications. Given the significant differences in meeting guidelines across age, sex, race, and BMI, PA interventions should account for these disparities during implementation. Given that our sample consists of individuals who already own fitness trackers, a promising avenue for PA interventions is a mobile health (mHealth) approach. By harnessing digital technologies, mHealth has the potential to enhance the accessibility, engagement, and personalization of PA interventions. Mobile platforms, such as apps and websites, can address key social determinants of health by overcoming geographical, logistical, and financial barriers. These platforms offer continuous support and near real-time monitoring of PA and factors that could impact one’s activity levels (eg, stress levels, mood, and symptom burden), providing interventionists with more accurate and near real-time PA tracking to tailor prescriptions [25,68,69]. mHealth solutions can customize the content, include behavior change techniques into their functionality, and provide exercise recommendations to meet individual needs, potentially boosting engagement and effectiveness [25,69-71]. This approach could be used to intervene in the sociodemographic groups that were less likely to adhere to the 2018 PAGA guidelines (ie, women, higher BMI individuals, non-Hispanic Black individuals, and those aged 30-49 or ≥70 years). Focusing these applications on those groups that would most benefit from increases in PA may lead to significant public health benefits.

Additionally, the algorithm used for estimating PA can have a significant effect on the accuracy of estimates. Thus, it is crucial for interventionists to recognize and adjust for potential systemic biases caused by different algorithms. Finally, health care systems should also be cautious about relying on “black box” algorithms, as the wide variability in results may interfere with decision-making accuracy. We are optimistic that the insights and results detailed in this paper will drive others to use more targeted and effective strategies to boost adherence to PA guidelines and use evidence-based methods for processing PA data.

## Appendix

Multimedia Appendix 1 CONSORT (Consolidated Standards of Reporting Trials) diagram.

Multimedia Appendix 2 Minutes per week (means and SEs) of sedentary, light-, moderate-, and vigorous-intensity physical activity among US adults by sex, age group, ethnicity, and BMI according to various algorithms.

## Abbreviations

AOR: adjusted odds ratio
AoU: All of Us
CONSORT: Consolidated Standards of Reporting Trials
HR: heart rate
LPA: light-intensity aerobic physical activity
mHealth: mobile health
MPA: moderate-intensity aerobic physical activity
MVPA: moderate- and vigorous-intensity aerobic physical activity
NCHS: National Center for Health Statistics
NIH: National Institutes of Health
OR: odds ratio
PA: physical activity
PAGA: Physical Activity Guidelines for Americans
VPA: vigorous-intensity aerobic physical activity

## Data Availability

The datasets generated and analyzed during this study are available from the corresponding author on reasonable request. This study used data from the All of Us Research Program’s Controlled Tier Dataset (version 7), available to authorized users on the Researcher Workbench.
