# Supplementary figures and images for: Analysis of Physical Activity Using Wearable Health Technology in US Adults Enrolled in the All of Us Research Program: Multiyear Observational Study
Source: J Med Internet Res. 2024 Dec 10;26:e65095. doi: 10.2196/65095 (PMC11668988; doi:10.2196/65095)

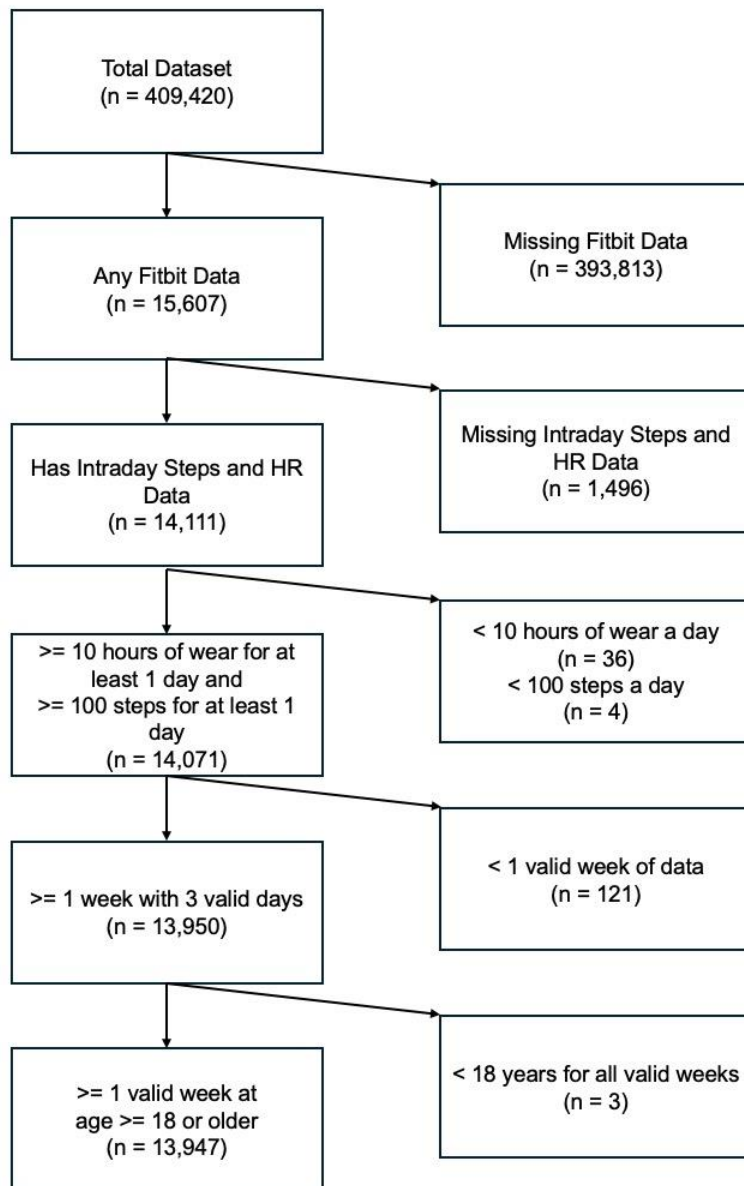

Supplement: Multimedia Appendix 1 [file jmir_v26i1e65095_app1.pdf]
