# Supplementary material for: Analysis of Physical Activity Using Wearable Health Technology in US Adults Enrolled in the All of Us Research Program: Multiyear Observational Study
Source: J Med Internet Res. 2024 Dec 10;26:e65095. doi: 10.2196/65095 (PMC11668988; doi:10.2196/65095)
Supplement: Multimedia Appendix 2 [file jmir_v26i1e65095_app2.pdf]

Table S1. Minutes per week (means and SEs) of sedentary, light, moderate, and vigorous intensity physical activity among U.S. adults by gender, age group, ethnicity, and BMI according to Fitbit algorithm.[a]

		Sedentary (1)		LPA (1)		MPA (1)		VPA (1)	
	N	Mean	SE	Mean	SE	Mean	SE	Mean	SE
**Total**	13,947	4,665.6	7.7	1,381.8	3.7	115.5	0.9	134.3	1.2
**Sex**									
Male	4,007	4,687.6	14.2	1,327.0	6.3	161.0	1.9	203.1	2.7
Female	9,553	4,656.9	9.3	1,405.0	4.6	96.2	0.9	105.8	1.1
Other / Not Specified	387	4,650.9	47.4	1,379.0	21.9	120.6	5.4	126.2	6.1
**Race/Ethnicity**									
Hispanic	310	4,510.2	48.0	1,433.3	27.6	116.2	5.6	121.9	6.6
Non-Hispanic White	11,109	4,669.6	8.7	1,383.7	4.1	116.6	0.9	137.5	1.3
Non-Hispanic Black	684	4,824.3	34.0	1,368.0	16.8	99.6	4.1	108.1	6.0
Non-Hispanic Asian / Pac. Islander	430	4,718.2	40.2	1,322.7	19.4	118.7	4.8	147.5	6.0
2 or More Races	293	4,613.5	50.0	1,362.9	23.3	108.2	6.2	114.3	6.4
Other / Not Specified	1,121	4,565.1	26.2	1,384.8	12.9	114.6	3.2	122.5	3.7
**BMI[b]**									
18 – 24.9	1,994	4,632.1	20.5	1,473.7	9.1	132.4	2.3	169.9	3.6
25 – 29.9	2,487	4,728.2	17.8	1,424.0	8.3	127.1	2.1	156.4	2.9
30 – 34.9	1,501	4,800.5	21.9	1,382.9	10.5	107.5	2.3	121.8	3.0
≥ 35	1,375	4,824.6	21.8	1,310.0	10.9	95.2	2.3	90.0	2.5
Other / Not Specified	6,590	4,588.2	11.5	1,352.9	5.6	112.1	1.3	127.3	1.7
**Age**									
18 – 29	1,511	4,364.2	20.0	1,374.7	10.7	107.6	2.1	111.7	2.5
30 – 39	2,528	4,453.8	15.7	1,457.4	9.0	108.5	2.0	111.8	2.2
40 – 49	2,259	4,606.9	18.2	1,447.1	9.3	105.1	2.0	114.4	2.4
50 – 59	2,760	4,736.5	17.5	1,397.3	8.2	116.5	2.0	134.6	2.8
60 – 69	3,102	4,806.5	17.5	1,361.5	7.6	128.2	2.0	159.9	2.7
≥ 70	1,787	4,939.8	22.3	1,210.0	9.1	121.8	2.4	165.4	3.8

[a] Table shows mean weekly minutes of MVPA, where MVPA is defined as minutes of moderate activity + 2 * minutes of vigorous activity. Only activity bouts ≥ 10 minutes in duration included in average figures.

[b] BMI was calculated as the average of all measured values measured between the first and last valid weeks for each participant. If no measured values were available, BMI was categorized as "Other / Not Specified". Individuals with a BMI under 18.5 are categorized as "Other / Not Specified" due to the small sample size.

Table S2. Minutes per week (means and SEs) of sedentary, light, moderate, and vigorous intensity physical activity among U.S. adults by gender, age group, ethnicity, and BMI according to HR derived metrics and counting only bouts ≥ 10 minutes.[a]

		Sedentary		LPA		MPA		VPA	
	N	Mean	SE	Mean	SE	Mean	SE	Mean	SE
**Total**	13,947	5,259.0	9.7	574.4	3.8	126.0	1.6	20.3	0.3
**Sex**									
Male	4,007	5,404.4	17.5	522.9	6.6	128.1	3.1	25.5	0.8
Female	9,553	5,200.0	11.9	592.5	4.6	123.7	2.0	18.2	0.4
Other / Not Specified	387	5,209.9	56.9	660.3	26.0	159.7	12.7	18.8	1.6
**Race/Ethnicity**									
Hispanic	310	5,358.7	64.4	494.2	22.6	79.2	7.0	15.9	1.8
Non-Hispanic White	11,109	5,303.1	10.7	576.9	4.2	129.5	1.9	20.7	0.4
Non-Hispanic Black	684	4,770.2	46.3	666.0	18.6	132.9	7.8	20.4	1.9
Non-Hispanic Asian / Pac. Islander	430	5,366.7	55.0	467.6	18.1	92.9	7.0	23.0	1.8
2 or More Races	293	5,360.9	64.6	458.7	22.1	75.3	7.1	15.6	1.6
Other / Not Specified	1,121	5,024.4	36.7	586.6	14.0	125.6	6.3	17.4	0.9
**BMI[b]**									
18 – 24.9	1,994	5,413.4	23.7	558.0	8.8	148.1	4.6	32.1	1.3
25 – 29.9	2,487	5,310.7	21.7	593.2	8.6	143.1	4.0	21.9	0.7
30 – 34.9	1,501	5,309.3	28.4	615.5	11.9	123.6	4.8	15.1	0.7
≥ 35	1,375	5,157.0	30.9	605.7	12.5	101.0	4.3	9.8	0.5
Other / Not Specified	6,590	5,202.6	14.8	556.4	5.6	118.6	2.4	19.5	0.5
**Age**									
18 – 29	1,511	5,374.0	28.7	348.9	7.4	33.9	1.6	12.8	0.6
30 – 39	2,528	5,408.6	23.0	428.1	7.2	47.4	1.6	13.9	0.5
40 – 49	2,259	5,292.1	23.9	565.4	9.2	85.1	3.0	15.5	0.6
50 – 59	2,760	5,169.5	22.1	636.4	8.9	137.6	3.8	19.8	0.8
60 – 69	3,102	5,166.3	20.6	694.3	8.2	193.7	4.1	26.0	0.8
≥ 70	1,787	5,207.4	27.0	679.4	11.1	230.9	6.1	32.4	1.4

[a] Table shows mean weekly minutes of MVPA, where MVPA is defined as minutes of moderate activity + 2 * minutes of vigorous activity. Only activity bouts ≥ 10 minutes in duration included in average figures.

[b] BMI was calculated as the average of all measured values measured between the first and last valid weeks for each participant. If no measured values were available, BMI was categorized as "Other / Not Specified". Individuals with a BMI under 18.5 are categorized as "Other / Not Specified" due to the small sample size.

Table S3. Minutes per week (means and SEs) of sedentary, light, moderate, and vigorous intensity physical activity among U.S. adults by gender, age group, ethnicity, and BMI according to step intensity derived metrics and counting only bouts ≥ 10 minutes.[a]

		Sedentary		LPA		MPA		VPA	
	N	Mean	SE	Mean	SE	Mean	SE	Mean	SE
**Total**	13,947	6,302.4	6.9	183.9	1.0	39.9	0.5	7.8	0.3
**Sex**									
Male	4,007	6,338.1	12.7	226.1	2.0	47.0	1.1	11.3	0.6
Female	9,553	6,285.1	8.5	166.6	1.0	37.1	0.6	6.5	0.2
Other / Not Specified	387	6,360.0	39.9	172.8	5.3	37.2	3.1	4.8	0.9
**Race/Ethnicity**									
Hispanic	310	6,261.6	47.9	181.4	6.1	30.9	2.7	5.9	1.0
Non-Hispanic White	11,109	6,340.4	7.5	184.7	1.1	42.0	0.6	8.0	0.3
Non-Hispanic Black	684	6,059.0	35.2	162.7	4.3	26.3	2.3	5.9	1.2
Non-Hispanic Asian / Pac. Islander	430	6,255.1	41.2	213.9	4.9	44.6	2.8	11.9	1.5
2 or More Races	293	6,238.1	47.6	180.4	6.0	26.0	1.9	7.5	1.5
Other / Not Specified	1,121	6,120.5	27.3	178.3	3.2	32.4	1.5	5.8	0.6
**BMI**[b]									
18 – 24.9	1,994	6,410.9	15.9	220.4	2.5	60.1	1.8	14.2	1.0
25 – 29.9	2,487	6,379.7	14.6	203.4	2.2	46.8	1.3	8.0	0.5
30 – 34.9	1,501	6,403.6	19.4	170.7	2.6	33.4	1.3	3.9	0.4
≥ 35	1,375	6,281.3	21.8	140.4	2.5	18.3	0.9	1.5	0.2
Other / Not Specified	6,590	6,221.8	10.9	177.5	1.4	37.2	0.7	8.0	0.4
**Age**									
18 – 29	1,511	6,088.0	21.9	193.7	2.6	32.0	1.1	7.9	0.5
30 – 39	2,528	6,214.7	17.1	185.1	2.1	28.1	0.9	8.2	0.5
40 – 49	2,259	6,288.5	16.9	176.9	2.2	31.8	1.0	8.6	0.6
50 – 59	2,760	6,294.8	15.5	186.5	2.2	40.1	1.2	8.5	0.7
60 – 69	3,102	6,398.4	14.1	189.3	2.1	52.1	1.3	8.0	0.6
≥ 70	1,787	6470.7	18.0	169.1	2.8	52.3	1.9	4.9	0.6

[a] Table shows mean weekly minutes of MVPA, where MVPA is defined as minutes of moderate activity + 2 * minutes of vigorous activity. Only activity bouts ≥ 10 minutes in duration included in average figures.

[b] BMI was calculated as the average of all measured values measured between the first and last valid weeks for each participant. If no measured values were available, BMI was categorized as "Other / Not Specified". Individuals with a BMI under 18.5 are categorized as "Other / Not Specified" due to the small sample size.

Table S4. Minutes per week (means and SEs) of sedentary, light, moderate, and vigorous intensity physical activity among U.S. adults by gender, age group, ethnicity, and BMI according to HR derived metrics and counting all bouts.[a]

		**Sedentary**		**LPA**		**MPA**		**VPA**	
	N	Mean	SE	Mean	SE	Mean	SE	Mean	SE
**Total**	13,947	5,259.0	9.7	428.6	2.9	260.6	2.4	31.5	0.4
**Sex**									
Male	4,007	5,404.4	17.5	382.7	5.1	255.6	4.4	38.2	0.9
Female	9,553	5,200.0	11.9	445.1	3.6	260.6	2.9	28.7	0.4
Other / Not Specified	387	5,209.9	56.9	496.4	20.3	311.6	17.8	30.9	2.1
**Race/Ethnicity**									
Hispanic	310	5,358.7	64.4	380.3	18.0	184.5	11.6	24.4	2.2
Non-Hispanic White	11,109	5,303.1	10.7	428.3	3.2	266.7	2.7	32.1	0.5
Non-Hispanic Black	684	4,770.2	46.3	507.3	14.7	279.7	11.1	32.3	2.4
Non-Hispanic Asian / Pac. Islander	430	5,366.7	55.0	349.1	13.9	200.4	11.1	33.9	2.2
2 or More Races	293	5,360.9	64.6	357.0	17.6	169.5	11.4	23.1	1.9
Other / Not Specified	1,121	5,024.4	36.7	446.5	11.0	255.5	8.9	27.6	1.1
**BMI**[b]									
18 – 24.9	1,994	5,413.4	23.7	395.7	6.4	295.7	6.8	46.9	1.5
25 – 29.9	2,487	5,310.7	21.7	432.0	6.6	291.3	5.9	34.9	0.9
30 – 34.9	1,501	5,309.3	28.4	464.1	9.3	264.4	7.2	25.7	0.9
≥ 35	1,375	5,157.0	30.9	472.6	10.0	226.4	6.6	17.6	0.6
Other / Not Specified	6,590	5,202.6	14.8	420.1	4.4	244.6	3.5	29.7	0.6
**Age**									
18 – 29	1,511	5,374.0	28.7	282.2	6.2	95.4	2.7	18.1	0.7
30 – 39	2,528	5,408.6	23.0	343.2	5.9	126.5	2.8	19.8	0.6
40 – 49	2,259	5,292.1	23.9	440.1	7.3	203.2	4.5	22.8	0.6
50 – 59	2,760	5,169.5	22.1	473.8	7.0	289.7	5.5	30.4	0.9
60 – 69	3,102	5,166.3	20.6	497.2	6.4	375.3	5.8	41.5	1.0
≥ 70	1,787	5,207.4	27.0	470.1	8.6	418.2	8.4	54.4	1.7

[a] Table shows mean weekly minutes of MVPA, where MVPA is defined as minutes of moderate activity + 2 * minutes of vigorous activity. All activity bouts (including those ≤ 10 minutes in duration) included in average figures.

[b] BMI was calculated as the average of all measured values measured between the first and last valid weeks for each participant. If no measured values were available, BMI was categorized as “Other / Not Specified”. Individuals with a BMI under 18.5 are categorized as “Other / Not Specified” due to the small sample size.

Table S5. Minutes per week (means and SEs) of sedentary, light, moderate, and vigorous intensity physical activity among U.S. adults by gender, age group, ethnicity, and BMI according to step intensity derived metrics and counting all bouts.[a]

		Steps		Sedentary		LPA		MPA		VPA	
	N	Mean	SE	Mean	SE	Mean	SE	Mean	SE	Mean	SE
**Total**	13,947	47,362	189.1	6,302.4	6.9	140.5	0.7	80.8	0.7	10.3	0.3
**Sex**											
Male	4,007	53,111	377.5	6,338.1	12.7	178.0	1.5	92.6	1.5	13.8	0.7
Female	9,553	45,021	217.4	6,285.1	8.5	125.0	0.8	76.2	0.8	9.0	0.3
Other / Not Specified	387	45,631	1053.8	6,360.0	39.9	134.2	4.0	73.8	4.0	6.7	1.0
**Race/Ethnicity**											
Hispanic	310	45,942	1135.8	6,261.6	47.9	134.3	4.3	75.4	4.0	8.4	1.1
Non-Hispanic White	11,109	47,858	214.2	6,340.4	7.5	141.8	0.8	82.5	0.8	10.5	0.3
Non-Hispanic Black	684	42,793	906.4	6,059.0	35.2	125.9	3.1	60.4	3.3	8.5	1.4
Non-Hispanic Asian / Pac. Islander	430	49,342	964.3	6,255.1	41.2	151.4	3.7	103.0	3.8	16.0	1.6
2 or More Races	293	44,487	1133.9	6,238.1	47.6	133.9	4.3	70.0	3.5	9.9	1.6
Other / Not Specified	1,121	45,622	616.6	6,120.5	27.3	135.8	2.4	72.5	2.2	8.1	0.7
**BMI**[b]											
18.5 – 24.9	1,994	55,841	518.1	6,410.9	15.9	161.4	1.9	115.0	2.3	18.4	1.1
25 – 29.9	2,487	51,479	442.3	6,379.7	14.6	156.1	1.7	91.6	1.7	10.6	0.6
30 – 34.9	1,501	44,820	492.9	6,403.6	19.4	133.4	2.0	68.8	1.8	5.7	0.5
≥ 35	1,375	38,009	461.4	6,281.3	21.8	112.2	2.0	45.5	1.4	2.5	0.3
Other / Not Specified	6,590	45,773	279.7	6,221.8	10.9	135.8	1.1	76.5	1.0	10.4	0.4
**Age**											
18 – 29	1,511	45,545	475.5	6,088.0	21.9	135.8	1.8	86.8	1.9	11.0	0.6
30 – 39	2,528	46,882	410.2	6,214.7	17.1	138.5	1.6	72.1	1.4	10.8	0.6
40 – 49	2,259	46,939	445.6	6,288.5	16.9	136.4	1.7	70.0	1.4	10.9	0.6
50 – 59	2,760	48,501	462.9	6,294.8	15.5	144.6	1.7	79.6	1.7	10.9	0.8
60 – 69	3,102	49,668	425.6	6,398.4	14.1	148.3	1.7	90.5	1.7	10.5	0.6
≥ 70	1,787	44,350	540.8	6,470.7	18.0	132.5	2.1	86.9	2.4	7.0	0.7

[a] Table shows mean weekly minutes of MVPA, where MVPA is defined as minutes of moderate activity + 2 * minutes of vigorous activity. All activity bouts (including those ≤ 10 minutes in duration) included in average figures.

[b] BMI was calculated as the average of all measured values measured between the first and last valid weeks for each participant. If no measured values were available, BMI was categorized as "Other / Not Specified". Individuals with a BMI under 18.5 are categorized as "Other / Not Specified" due to the small sample size.
